# Effects of Naphthazarin (DHNQ) Combined with Lawsone (NQ-2-OH) or 1,4-Naphthoquinone (NQ) on the Auxin-Induced Growth of *Zea mays* L. Coleoptile Segments

**DOI:** 10.3390/ijms20071788

**Published:** 2019-04-11

**Authors:** Małgorzata Rudnicka, Michał Ludynia, Waldemar Karcz

**Affiliations:** Department of Plant Physiology, Faculty of Biology and Environmental Protection, University of Silesia, Jagiellońska 28, PL-40032 Katowice, Poland; malgorzata.rudnicka@us.edu.pl (M.R.); michal.ludynia@us.edu.pl (M.L.)

**Keywords:** auxin, biopesticide, synergistic effect, lawsone, 1,4-naphthoquinone, naphthazarin

## Abstract

Naphthoquinones, plants secondary metabolites are known for their antibacterial, antifungal, anti-inflammatory, anti-cancer and anti-parasitic properties. The biological activity of naphthoquinones is connected with their ability to generate reactive oxygen species and to modify biological molecules at their nucleophilic sites. In our research, the effect of naphthazarin (DHNQ) combined with 2-hydroxy-1,4-naphthoquinone (NQ-2-OH) or 1,4-naphthoquinone (1,4-NQ) on the elongation growth, pH changes of the incubation medium, oxidative stress and redox activity of maize coleoptile cells were investigated. This paper describes experiments performed with maize (*Zea mays* L.) coleoptile segments, which is a classical model system to study plant cell elongation growth. The data presented clearly demonstrate that lawsone and 1,4-naphthoquinone combined with naphthazarin, at low concentrations (1 and 10 nM), reduced the endogenous and IAA-induced (Indole-3-Acetic Acid) elongation growth of maize coleoptile segments. Those changes in growth correlated with the proton concentration in the incubation medium, which suggests that the changes in the growth of maize coleoptile segments observed in the presence of naphthoquinones are mediated through the activity of PM H^+^-ATPase. The presence of naphthoquinones induced oxidative stress in the maize coleoptile tissue by producing hydrogen peroxide and causing changes in the redox activity. Moreover, the incubation of maize segments with both naphthoquinones combined with naphthazarin resulted in lipid peroxidation and membrane damage. The regulation of PM H^+^-ATPase activity, especially its inhibition, may result from two major types of reaction: first, a direct interaction between an enzyme and naphthoquinone, which leads to the covalent modification of the protein thiols and the generation of thioethers, which have been found to alter the activity of the PM H^+^-ATPases; second, naphthoquinones induce reactive oxygen species (ROS) production, which inhibits PM H^+^-ATPases by increasing cytosolic Ca^2+^. This harmful effect was stronger when naphthazarin and 1,4-naphthoquinone were added together. Taking these results into account, it can be suggested that by combining naphthoquinones in small quantities, an alternative to synthetic pesticides could be developed.

## 1. Introduction

Phytohormones are plant substances that have a high biological activity at very low concentrations. They function as signaling molecules that support the development, abiotic and biotic stress responses and many other physiological processes in plants. The major classes of phytohormones are auxins, cytokinins, gibberellins, brassinolides, jasmonates, abscisic acid, ethylene, strigolactone and salicylic acid. The first identified and isolated phytohormone was auxin (Indole-3-Acetic Acid, IAA), which was determined to be responsible for the light-induced differential elongation in grass coleoptiles (reviewed in [1,2]). Auxins are one of the most multi-functional phytohormones as they are vital for cell division, plant growth and development, polarity, apical dominance and responses to pathogens and abiotic stress [3,4,5]. Moreover, auxins interact with many other phytohormones [6].

Auxin action can be divided into two types of responses: non-transcriptional and transcriptional. This first type includes very fast reactions such as the activation of the plasma membrane proton pumps, transient PM depolarisation and subsequent hyperpolarisation, the activation of ion channels such as the K^+^- and Cl^−^-uptake channels and cortical microtubule reorganisation [7,8,9,10,11].

Transcriptional auxin responses are mediated through the regulation of the gene expression. This process requires three molecular components: Aux/IAA repressors, receptors of the TIR1/AFB protein family and auxin response factors. Auxin enhances the binding of Aux/IAA proteins to the TIR1 receptor complex, which subsequently triggers the degradation of these transcriptional repressors. The removal of Aux/IAAs enables the auxin response factors (ARFs) to activate the transcription of the auxin-responsive genes [12,13,14,15].

Naphthoquinones, which are naphthalene derivatives, are natural phenolic compounds that consist of two rings that are fused together, namely benzene and quinone, in which the carbonyl groups are in the para position. These compounds, produced by bacteria, fungi and higher plants (*Bignoniaceae, Droseraceae, Ebenaceae, Juglandaceae* and *Plumbaginaceae*), are widely distributed [16,17,18,19]. Naphthoquinones as secondary metabolites, are a group of highly reactive molecules that act on a broad spectrum of cell components and they probably play a protective role against herbivores. Quinones indirectly reduce the nutritional value of plant components for herbivores through the alkylation of proteins or through interactions with other organic molecules. Quinones might also have a direct toxic effect on the insects that attack plants by initiating the production of reactive oxygen species [20,21,22].

The toxic effect of naphthoquinones is connected with the amount, type and location of substituents (e.g., hydroxyl or methyl groups) in their molecules. There are two general action mechanisms of naphthoquinones: direct, in which naphthoquinone molecules are involved in the process of protein modification, and indirect, in which the mediators are reactive oxygen species, which oxidize various structures in the cell [23,24].

Lawsone is a characteristic compound in the leaves and flowers of *Lawsonia inermis* L. [25]. An intermediate, 1,4-naphthoquinone can be found, for example, in black walnut leaves. Naphthazarin is one of the secondary metabolites derived from the members of the *Boraginaceae, Droseraceae* and *Nepenthaceae* families [16,18]. Naphthoquinones have become very popular in recent years because of their properties and their applicability in, for example, the pharmaceutical, energy or chemical industries. It has been shown that 1,4-naphthoquinone and lawsone also have anti-corrosive properties. These compounds cover metal with a thin film, which reduces the formation of corrosion pits by more than 90% [26,27]. In addition, lawsone and its derivatives can be used to effectively fight the parasitic disease – schistosomiasis. These substances interrupt the life cycle of *Schistosoma mansoni* by eradicating the aquatic gastropod mollusk *Biomphalaria glabrata*, which is its intermediate host [28]. Furthermore, lawsone, naphthazarin and other naphthoquinones and their derivatives can serve as the core for a new class of drugs that are used to treat diseases such as diabetes, cancer, metabolic syndromes, insulin resistance, as well as being used for immunoregulation [29,30,31,32]. Lawsone can also be used as a colorimetric and electrochemical sensor for industrial waste such as cyanides, acetates or fluorine [33]. Naphthazarin and lawsone molecules and their derivatives can additionally be used in eco-batteries because of their high redox properties [34,35]. However, in this work, the most interesting use of naphthoquinones is as alternative environmentally friendly agents for protecting plants.

The current status of research on environmentally friendly natural plant products suggests that they really are alternatives that can replace synthetic pesticides. In contrast to synthetic pesticides, biopesticides decompose quickly without contaminating the soil and are effective in small quantities. It is possible that by combining these natural plant products in small quantities, an economically viable alternative to synthetic pesticides can be developed.

The main goal of our research was to investigate the mechanisms by which naphthazarin combined with lawsone or 1,4-naphthoquinone causes changes in the IAA-induced growth of plant cells. This goal was realised by (1) studying the effect of naphthazarin (DHNQ, 5,8-dihydroxy-1,4-naphthoquinone) combined with lawsone (NQ-2-OH, 2-hydroxy-1,4-naphthoquinone) or 1,4-naphthoquinone (NQ), all at low concentrations (1 and 10 nM), on the endogenous and IAA-induced growth of maize coleoptile segments and the pH of a medium, which was measured simultaneously with the growth; (2) establishing the effect of DHNQ combined with NQ-2-OH or NQ on the H_2_O_2_ production and plasma membrane redox activity and (3) examining the effect of DHNQ and its combination with NQ-2-OH or NQ on the malondialdehyde (MDA) content of coleoptile segments and on the membrane integrity and cell viability in maize coleoptile segments. The results presented demonstrate that both lawsone and 1,4-naphthoquinone combined with naphthazarin at low concentrations reduce the endogenous and IAA-induced growth of maize coleoptile segments, which may be connected with the contribution of these secondary metabolites to a direct interaction with PM H^+^-ATPases, ROS production and membrane damage. 

## 2. Results 

### 2.1. Effects of Naphthazarin (DHNQ) Combined with Lawsone (NQ-2-OH) or 1,4-Naphthoquinone (NQ) on the Endogenous and Auxin-Induced Growth of Maize Coleoptile Segments

**Figure 1:**
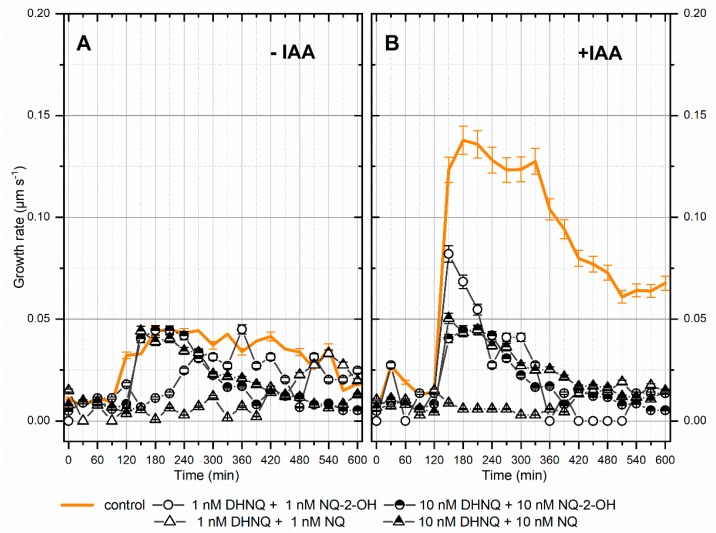
The effect of DHNQ combined with NQ-2-OH or NQ on the growth rate (µm s^−1^) of the maize coleoptile segments that had been incubated (**A**) without and (**B**) with IAA. The coleoptile segments were preincubated (over 1 h) in a control medium, after which the naphthoquinones were added. IAA was added to the incubation medium at 2 h. For the sake of clarity, the data were shown in 30 min intervals. The presented data is the mean of six independent experiments. Bars indicate the means ± SEs.

In general, the incubation of maize coleoptile segments with naphthazarin (DHNQ) combined with lawsone (NQ-2-OH) or 1,4-naphthoquinone (NQ), at concentrations of both 1 nM and 10 nM, caused a decrease in the endogenous and auxin-induced growth rate of the coleoptile segments (Figure 1A,B). Considering the effect of naphthazarin combined with lawsone or 1,4-naphthoquinone on the endogenous growth, the administration of both combinations at a concentration of 1 nM caused a strong, fast and long-term inhibition of the growth rate, especially for the combination of naphthazarin and 1.4-naphthoquinone. Although the higher concentration of both combinations also had a harmful effect on the growth rate of maize coleoptile segments, this occurred with a delay. When IAA was added to the control medium (2 h after the start of the experiment), there was a strong increase in the growth rate with a maximal growth rate of 0.14 µm s^−1^ (Figure 1B), compared to the control. The kinetics of the IAA-induced growth rate of the coleoptile segments was disturbed for all of the tested treatments. The highest decrease of the auxin-induced growth rate, was recorded for the combination of naphthazarin and 1,4-naphthoquinone at a concentration of 10 nM (Figure 1B).

**Figure 2:**
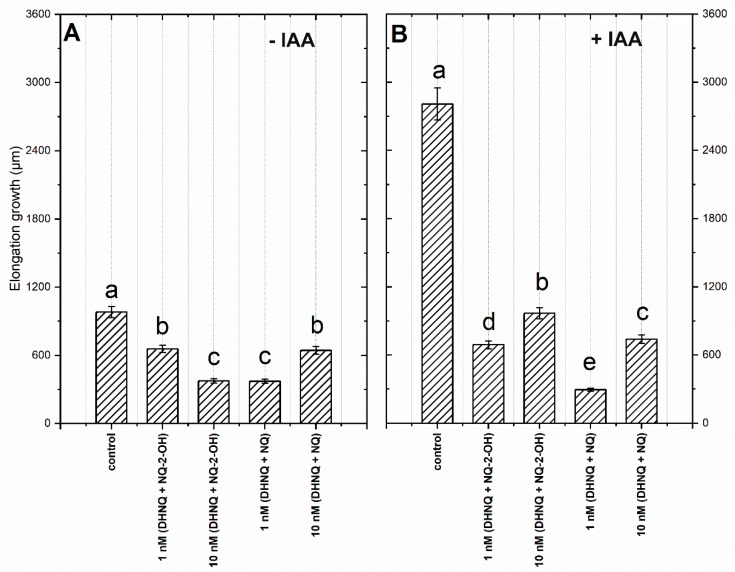
The effect of DHNQ combined with NQ-2-OH or NQ on the cumulative growth of the maize coleoptile segments that were incubated without (**A**) and with IAA (**B**). The figure shows the differences between the cumulative growth per coleoptile segment at 600 and 120 min. Mean values that are followed by the same letter within a group were not significantly different from each other. The presented data is the mean of six independent experiments. Bars indicate the means ± SEs.

To determine the effect of naphthazarin combined with lawsone or 1,4-naphthoquinone on the elongation growth, the cumulative growth was calculated as the sum of the extensions measured at three-minute intervals over 10 h (Figure 2). The strongest inhibition of endogenous growth of more than 62% was observed for the 1 nM combination of naphthazarin and 1,4-naphthoquinone (to 371.9 ± 18.6 µm in comparison to the growth in the control medium of 981.7 ± 49.1 µm) and 61.6% for the combination of DHNQ and NQ-2-OH at 10 nM (376.6 ± 18.9 µm) (Figure 2A). A slight decrease (of 35%) was observed for DHNQ and NQ-2-OH at 1 nM and for the combination of 10 nM of DHNQ and NQ. The cumulative IAA-induced elongation of the maize coleoptile segments was approximately 2.8-fold greater than for the control (Figure 2B).

When naphthazarin (DHNQ) combined with lawsone (NQ-2-OH) or 1,4-naphthoquinone (NQ) was added to a medium, there was a decrease in the IAA-induced growth of the maize coleoptile segments of more than 60% (Figure 2B). Interestingly, the most detrimental combination of all of the tested treatments was naphthazarin combined with 1,4-naphthoquinone at 1 nM, which reduced the IAA-induced growth by ca. 90%. 

Moreover, in order to compare the effects of naphthazarin combined with lawsone or 1,4-naphthoquinone on the endogenous and IAA-induced cumulative growth, a t-test was performed. This statistical analysis showed that there were statistically significant differences between the endogenous and auxin-induced growth of the maize coleoptile segments in all of the treatments that were studied. Taking the results into account, it can be suggested that in the presence of IAA, the mechanism of action of naphthoquinones applied together can complement each other and have a more negative effect. 

### 2.2. Proton Concentration in the Incubation Medium of the Coleoptile Segments 

**Figure 3:**
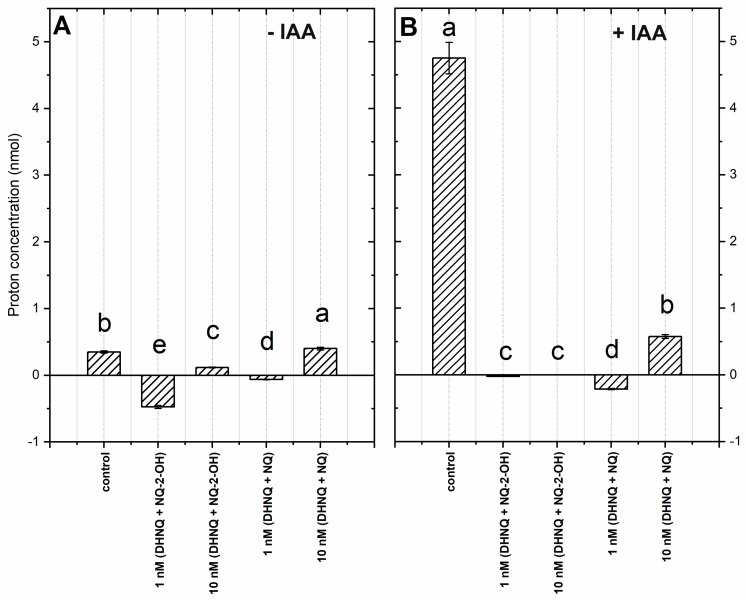
The effect of DHNQ combined with NQ-2-OH or NQ on the proton extrusion of the maize coleoptile segments that were incubated (**A**) without and (**B**) with IAA. The presented data show the differences between the H^+^ concentration per coleoptile segment at 600 and 120 min and are the mean of six independent experiments. Mean values that are followed by the same letter within a group were not significantly different from each other. Bars indicate the means ± SEs.

The simultaneous measurements of the growth and pH of the external medium indicated that the coleoptile segments changed the pH of the medium. This characteristic change of pH of the incubation medium occurred when an increase of the pH from 5.8–5.9 to 6.0–6.2 during the first 2 h was initially recorded, followed by a slow decrease to a pH of approximately 5.6–5.7 after 10 h. When the IAA at a final concentration of 100 µM was added to the incubation medium 2 h after the start of the experiment, there was an additional decrease in the pH to ca. 4.8 after 10 h of experiment (for a comparison see Rudnicka et al., 2019). In order to present the pH changes in the medium much more suggestively, they are shown as changes in the H^+^ concentration per coleoptile segment (Figure 3).

The addition of DHNQ combined with NQ-2-OH or with NQ to the control medium caused a decrease in proton excretion (excluding DHNQ with NQ at 10 nM, Figure 3A). The highest inhibition of proton release was recorded in the combination of naphthazarin with lawsone at a 1 nM concentration, which could be connected to the inactivation of the proton pumps in the presence of both naphthoquinones (Figure 3A). 

When added to the medium, auxin stimulated proton extrusion (Figure 3B). In the presence of all of the tested combinations, the auxin-induced proton release was extremely reduced, especially when DHNQ with NQ was added. 

In order to examine the effects of naphthazarin combined with lawsone or 1,4-naphthoquinone on the proton extrusion of the maize coleoptile segments that had been incubated with or without IAA, the t-test was performed. This statistical analysis showed that in all of the treatments that were studied, there were significant differences between the proton concentrations, which suggests that all adequate tested treatments incompletely diminished the auxin effect on the proton excretion.

### 2.3. Effects of Naphthazarin (DHNQ) Combined with Lawsone (NQ-2-OH) or 1,4-Naphthoquinone (NQ) on Oxidative Stress, Measured as the Production of H_2_O_2_ and Redox Activity

**Figure 4:**
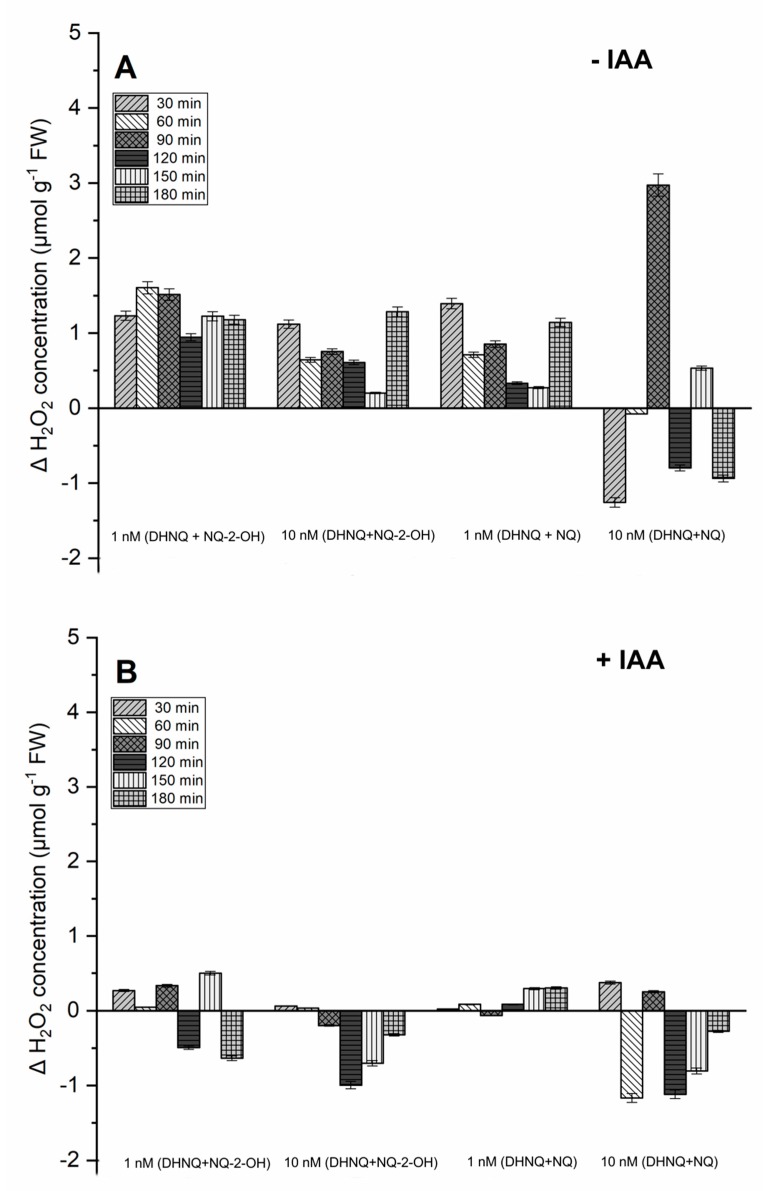
The effect of DHNQ combined with NQ-2-OH or NQ on hydrogen peroxide production in the maize coleoptile segments that had been incubated (**A**) without and (**B**) with IAA. The presented data is the mean of six independent experiments (delta received by the subtraction of the control value from the tested value). Bars indicate the means ± SEs.

The hydrogen peroxide production in the maize coleoptile segments that had been incubated in the control medium was low (2.23 ± 0.11 µmol g^−1^ FW) and increased only slightly during the experiment (data not shown). The administration of naphthazarin combined with lawsone at both of the tested concentrations caused a stronger increase in the H_2_O_2_ concentration than the combination of naphthazarin and 1,4-naphthoquinone (Figure 4A). Interestingly, this effect was not dependent on the concentration, and a higher amount of hydrogen peroxide was recorded with 1 nM of DHNQ combined with NQ-2-OH, especially during the first 120 min. A similar relation was observed in the case of the second combination that was tested (Figure 4A). 

The presence of auxin in the medium decreased the hydrogen peroxide production that was stimulated by the naphthoquinone treatment that was tested (Figure 4B). This effect was stronger in the case of the 10 nM concentration of both of the examined combinations. A 1 nM concentration of naphthazarin combined with lawsone or 1,4-naphthoquinone seemed to be more efficient in generating H_2_O_2_, and the presence of auxin only slightly decreased it.

**Figure 5:**
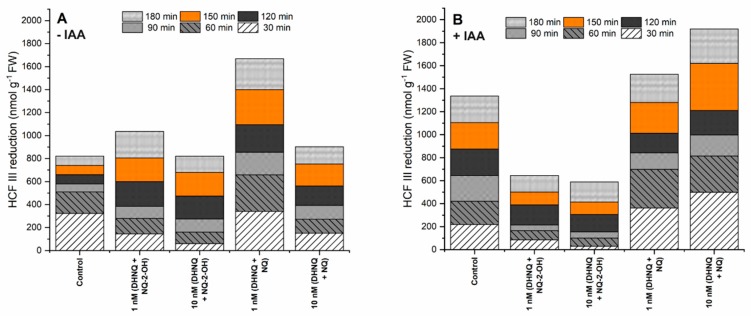
The effect of DHNQ combined with NQ-2-OH or NQ on the redox activity measured as the HCF III reduction in the maize coleoptile segments that had been incubated (**A**) without and (**B**) with IAA. The presented data is the mean of six independent experiments. Bars indicate the means ± SEs.

The redox activity, which was estimated as the hexacyanoferrate III (HCF III) reduction, in the control medium, increased during the first 60 min of the experiment, followed by a decrease and stabilisation of the redox activity to 821.9 ± 41.1 nmol g^−1^ FW after 180 min of the experiment (Figure 5A). The hexacyanoferrate III reduction strongly increased in the presence of naphthazarin combined with NQ at the 1 nM concentration. A similar but weaker response was observed when the maize coleoptile segments were incubated with 1 nM of naphthazarin and NQ-2-OH (Figure 5A). Conversely, both of the remaining tested combinations, naphthazarin combined with lawsone or 1,4-naphthoquinone at 10 nM, had only a minor effect on the redox activity, and the hexacyanoferrate III reduction levels were similar to the increase in the control conditions.

The addition of only auxin to the incubation medium caused an increase in the redox activity by ca. 60% (1336.2 ± 66.8 nmol g^−1^ FW) compared to the control (Figure 5B). The incubation of the maize coleoptile segments with the combination of DHNQ and NQ at concentrations of 1 and 10 nM in the presence of IAA caused a strong increase of the hexacyanoferrate III reduction (Figure 5B). However, the administration of auxin to the combination of naphthazarin and lawsone at both of the concentrations that were tested had the reverse effect on the redox activity in the maize coleoptile segments. In this case, the reduction of HCF III was diminished.

### 2.4. Effects of Naphthazarin (DHNQ) Combined with Lawsone (NQ-2-OH) or 1,4-Naphthoquinone (NQ) on Lipid Peroxidation and Cell Viability

**Figure 6:**
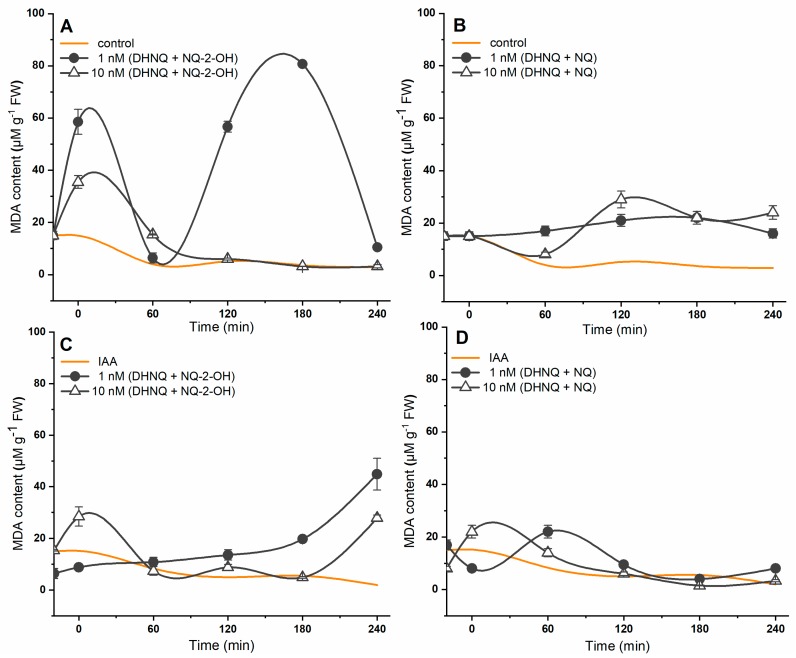
The effect of DHNQ combined with NQ-2-OH or NQ on lipid peroxidation, which was measured as the MDA content in the maize coleoptile segments that had been incubated (**A**,**B**) without and (**C**,**D**) with IAA. The presented data is the mean of six independent experiments. Bars indicate the means ± SEs.

The effect of naphthazarin combined with lawsone or 1,4-naphthoquinone on lipid peroxidation was also studied. The results are presented in Figure 6. In the control conditions, the MDA (Malondialdehyde) content decreased over the duration of the experiment. The introduction of auxin into the control medium only slightly affected the level of lipid peroxidation in the maize coleoptile segments. It was found that the incubation of the maize coleoptile segments with DHNQ and NQ-2-OH at concentrations of 1 and 10 nM caused an increase in the malondialdehyde (MDA) content (Figure 6A). Higher values of malondialdehyde were obtained for the combination of naphthazarin and lawsone at 1 nM. When auxin was present in the incubation medium, it caused a decrease in the lipid peroxidation of the maize coleoptile segments that had been incubated with both concentrations of naphthazarin combined with lawsone during the first 180 min of the experiments (Figure 6C). When the maize coleoptile cells were incubated with naphthazarin and 1,4-naphthoquinone, the MDA content was also increased compared to the control, but the effect was minor (Figure 6B). The presence of IAA reduced the malondialdehyde concentration in the maize coleoptile segments that had been incubated with a combination of DHNQ and NQ at 1 and 10 nM to values that were similar to the control (Figure 6D). Interestingly, when both combinations tested were compared, it was found that naphthazarin combined with lawsone induced a stronger lipid peroxidation in the maize coleoptile segments than the combination of DHNQ and NQ, especially at the 1 nM concentration.

**Figure 7:**
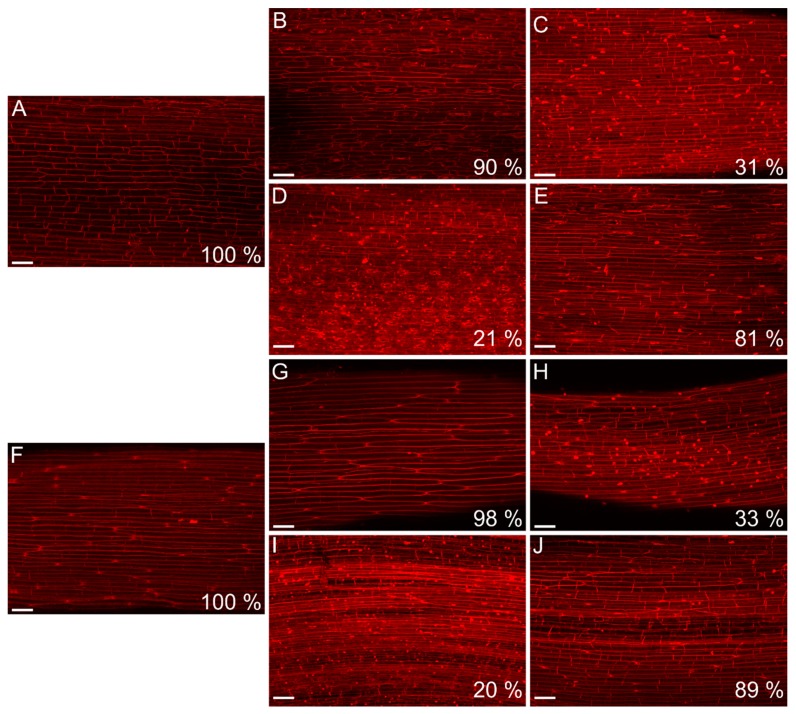
The effect of DHNQ combined with NQ-2-OH or NQ on the membrane integrity and cell viability in the maize coleoptile segments. (**A**) control medium, (**B**) 1 nM (DHNQ + NQ-2-OH); (**C**) 10 nM (DHNQ + NQ-2-OH); (**D**) 1 nM (DHNQ + NQ); (**E**) 10 nM (DHNQ + NQ); (**F**) 100 µM IAA; (**G**) 1 nM (DHNQ + NQ-2-OH) + IAA; (**H**) 10 nM (DHNQ + NQ-2-OH) + IAA; (**I**) 1 nM (DHNQ + NQ) + IAA; and (**J**) 10 nM (DHNQ + NQ) + IAA. The cell viability is presented as the percentage of living cells.

To determine the effect of DHNQ combined with NQ-2-OH or NQ on membrane damage, the viability of the maize coleoptile cells was detected using the propidium iodide (PI) method. The viability of the maize coleoptile cells after a 10 h incubation in the control medium and in the presence of IAA was at the same high level (Figure 7A,F). The incubation of the maize coleoptile cells with naphthazarin combined with lawsone or 1,4-naphthoquinone for 10 h caused major changes in the cell viability, excluding combination of DHNQ with NQ-2-OH at 1 nM and DHNQ with NQ at 10 nM (Figure 7B,E,G,J). The most significant decrease in the cell viability was observed for both the combination of 1 nM DHNQ and NQ and the combination of DHNQ and NQ-2-OH at a 10 nM concentration, independent of the presence of IAA (Figure 7C,D,H,I). A similar effect was recorded when the coleoptiles were incubated with 10 nM NQ-2-OH; however, in this case, the addition of IAA to the incubation medium reduced the membrane damaging effect of lawsone (Supplementary Materials 1E,K). Interestingly, the treatment of the maize coleoptile cells with DHNQ at 1 and 10 nM concentrations caused only minor changes in the cell viability after 10 h of the experiments, although the presence of IAA in the incubation medium containing DHNQ at 1 or 10 nM significantly reduced (more than 50%) the viability of the maize coleoptile cells (Supplementary Materials 1A,D,G,J).

## 3. Discussion

Naphthoquinones are a large group of natural compounds that are found in plants and fungi [18,36]. These substances are highly phytotoxic due to their ability to inhibit seed germination, plant respiration and growth [36,37,38,39]. The mechanisms of their activity have previously been investigated. For example, recently, it was shown that juglone, a naturally occurring naphthoquinone from walnut trees, inhibits IAA-induced growth and proton extrusion in maize coleoptile segments in a concentration-dependent manner [37]. This effect involves the inhibition of the plasma membrane H^+^-ATPase activity, which is irreversible. Moreover, as was shown in the paper by Rudnicka et al. [38], higher concentrations of lawsone and 1,4-naphthoquinone also have a negative effect on auxin-induced growth, proton extrusion and the hyperpolarisation of the membrane potential, parameters that are fundamental for the so-called “acid-growth hypothesis” of auxin action (for a review, see [39,40]). In addition, it was also found that juglone, lawsone and 1,4-naphthoquinone induced reactive oxygen species [38,41] and the subsequent programmed cell death [17]. Naphthoquinones are able to inhibit some enzymes and proteins and bind to DNA, thereby modifying its conformation [23,42,43].

Recently, it was found that DHNQ had a toxic effect on maize coleoptile segments by inhibiting endogenous and IAA-induced growth as well as proton extrusion. In the presence of naphthazarin at lower concentrations (<1 μM), there is an increase in hydrogen peroxide (H_2_O_2_) production, catalase activity, redox activity and malondialdehyde (MDA) content, which suggests a specific character of its action. It was also found that naphthazarin at concentrations higher than 0.1 μM caused the depolarisation of the membrane potential. The organisation of the cortical microtubules was also analysed, and it was shown that naphthazarin changed the IAA-induced microtubule reorientation. These results suggest that naphthazarin decreased the growth of maize coleoptile cells via a broad spectrum of effects [44].

This indicates that naphthoquinones act pleiotropically on plants, including the generation of reactive oxygen species (ROS), interactions with nucleophilic biomolecules and the inhibition of key proteins and enzymes. Because naphthoquinones can potentially be used as biopesticides [45], the main goal of this research was to determine the effect of naphthazarin combined with lawsone or 1,4-naphthoquinone, all at low concentrations, on auxin-induced elongation growth, proton extrusion, H_2_O_2_ production, catalase activity, lipid peroxidation and membrane integrity in maize coleoptile segments. 

It is generally agreed that the auxin-induced elongation growth of plant cells is primarily regulated by the plasma membrane (PM) H^+^-ATPases, which are sensitive to both structural and functional disturbances under stress conditions [46,47,48]. In order to examine the effect of those naphthoquinones on PM H^+^-ATPase at low concentrations, the growth reaction, oxidative stress level in maize coleoptile segments and pH of the segments’ medium were measured. The data obtained in this study indicate that the addition of IAA to the control medium alone increased the growth rate of the maize coleoptile segments (Figure 1B), increased the elongation growth (Figure 2B) and enhanced the proton extrusion (Figure 3B). These results are in agreement with previously obtained conclusions [11,49,50,51,52]. The incubation of maize coleoptile cells with naphthazarin (DHNQ) combined with lawsone (NQ-2-OH) or 1,4-naphthoquinone caused a strong decrease of both the endogenous and IAA-induced elongation growth in all of the treatments studied (Figure 2). The data presented in this paper clearly demonstrate that a combination of naphthazarin and 1,4-naphthoquinone at lower concentrations (1 nM) decreased the endogenous and auxin-induced elongation growth of the maize coleoptile cells in the most effective manner. However, naphthazarin combined with lawsone also decreased the elongation growth, especially at a concentration of 1 nM. A similar reaction was observed for proton extrusion, which was strongly inhibited in the presence of the combination of naphthazarin and 1,4-naphthoquinone at 1 nM, as well as in the presence of naphthazarin combined with lawsone at 1 and 10 nM. The correlation between the changes in growth and the proton concentration in the incubation medium of the coleoptile segments suggests that the changes in the growth of maize coleoptile segments are mediated via the PM H^+^-ATPase activity, which is in agreement with the “acid-growth hypothesis”. Interestingly, the strongest inhibition of the elongation growth and proton extrusion was observed for the combination of naphthoquinones at a lower concentration (1 nM). Although naphthazarin combined with 1,4-naphthoquinone had only a negligible effect on the hydrogen peroxide production and MDA content (Figure 4 and Figure 6), it caused a strong increase in the redox activity and cell viability at both of the concentrations that were tested (Figure 5 and Figure 7), regardless of the presence of auxin. In contrast, naphthazarin combined with lawsone induced an increase in the hydrogen peroxide production and lipid peroxidation of the maize coleoptile segments, but had only a minor effect on the redox activity at both of the concentrations studied. Moreover, DHNQ combined with lawsone at 10 nM exhibited a high level of membrane-disrupting activity, which was measured as the viability in the medium without IAA as well as in the presence of auxin (Figure 7C,H). 

The production of reactive oxygen species induced in the presence of the naphthoquinones that were tested may also cause membrane damage (Figure 7), which results in electrolyte leakage from the plant cells [53,54]. 

For the combination of naphthazarin with lawsone or 1,4-naphthoquinone, it should be pointed out that the inhibitory effect on the auxin-induced growth and proton extrusion of the maize coleoptile segments was stronger compared to the individual action of any of the naphthoquinones that were tested (see [38,44]). This synergetic effect was significantly more visible at a concentration of 1 nM, particularly when naphthazarin was combined with 1,4-naphthoquinone. Furthermore, in the presence of both naphthoquinones at 1 nM, the oxidative stress, which was measured as the H_2_O_2_ production, and the redox activity as well as the lipid peroxidation, was higher. Interestingly, regardless of the presence of auxin, naphthazarin and 1,4-naphthoquinone combined at a 1 nM concentration caused significant membrane damage. Taking into account all of the parameters that were studied, we suggest that the combination of both naphthoquinones at 1 nM had the strongest toxic effect. However, the question may be raised as to why the lower concentration of each naphthoquinones caused much stronger effects than the higher concentrations. The answer to this question is difficult, but we suppose that it may be connected to the sensitivity of cells, especially the gating mechanism of the ion channels (e.g., K^+^) to naphthoquinones. This suggestion could be supported by two facts: (1) that H_2_O_2_ induces K^+^ efflux which results in a dramatic K^+^ loss from plant cells [53]; and (2) that naphthoquinones block voltage gated potassium channels that are probably connected with both the oxidant nature and the structural determinants of naphthoquinone molecules [55]. Both of the facts may have a detrimental effect on the auxin-induced elongation growth of maize coleoptile cells. Our results suggest that the tested naphthoquinones have different modes of action in *Zea mays* coleoptile segments and that their combination might have a more harmful effect than when they are used separately.

Regulating the PM H^+^-ATPase activity, especially its inhibition, may result from two types of interactions. The first is the direct interaction between an enzyme and quinone, which leads to a covalent modification of the protein thiols and the generation of thioethers, and the second is the capacity of quinone to produce reactive oxygen species (ROS) [56,57]. Because auxin-induced growth is primarily regulated by PM H^+^-ATPase, which is sensitive to sulfhydryl reagents, it can be suggested that naphthazarin combined with lawsone or 1,4-naphthoquinone may inhibit the proton pump via a direct interaction with the enzyme thiols [58,59]. In contrast, it should be pointed out that the hydroxyl group at the C_2_ position in lawsone reduces the electrophilicity of C_3_ and also functions as a steric hindrance in the interactions with nucleophiles [23,24,56]. This fact suggests that lawsone inhibits auxin-induced growth indirectly by producing reactive oxygen species (ROS) that then increase cytosol Ca^2+^, which is followed by an inhibition of the PM H^+^-ATPase activity [60,61,62,63,64]. It was also found that lawsone has the ability to bind to DNA, which leads to a nonspecific interaction and the transition of Β-DNA into an A-DNA conformation [43].

The chemical structures of naphthazarin and 1,4-naphthoquinone as well as their ability to induce a nucleophilic attack on the biological structure may suggest that this naphthoquinone can affect the auxin nuclear-signaling system by inhibiting parvulin, which is the key enzyme responsible for the isomerisation of prolines in the AUX/IAA transcriptional repressors. This process is crucial for the interaction of AUX/IAA with the SCF-TIR1 and its degradation [65,66,67,68]. Furthermore, it was found that secondary metabolites such as juglone and naphthazarin [44] exhibit a high level of toxicity by affecting the polymerisation of the cortical microtubules, which are involved in the elongation of plant cells [69,70,71].

To summarise, the present study sheds new light on the effect of naphthazarin combined with lawsone or 1,4-naphthoquinone on the auxin-induced growth of maize coleoptile segments and suggests a herbicidal potency of their combinations due to their complementary mechanisms for inhibiting auxin-induced growth. 

## 4. Materials and Methods 

### 4.1. Plant Material

Seeds of maize (*Zea mays* L. cv. Cosmo 230) that had been soaked in tap water were sown on wet lignin in plastic boxes and placed in a growth chamber (Type MIR-553, Sanyo Electric Co., Osaka, Japan) at 27 ± 1.0 °C. After 96 h, ten-mm-long coleoptile segments were cut from the etiolated maize 3 mm below the tip, and the first leaf was removed. The coleoptile segments were incubated in a medium containing 1 mM KCl, 0.1 mM NaCl and 0.1 mM CaCl_2_ (control medium). The initial pH of the control medium was adjusted to 5.8–6.0 in all of the growth experiments.

### 4.2. Growth and pH Measurements

The elongation growth of the coleoptile segments and the pH of the incubation medium were simultaneously measured from the same tissue sample using a special apparatus [11,38,72] in which three glass pipettes that are connected via a silicon hose are filled with coleoptile segments that are placed vertically (20 segments in each). An angular position transducer (TWK-Electronik, Düsseldorf, Germany) was used to perform the high-resolution measurements of the growth rate of the coleoptile segments, which had been incubated in an intensively aerated medium. The medium flowed through the lumen of the cylinders, and this direct contact of the experimental solutions with the interior of the segments enhances the elongation growth of the coleoptile segments remarkably [73]. The pH of the incubation medium was measured using a pH electrode (OSH 10-10; Metron, Torun, Poland). All of the processes were performed under dim green light and at a thermostatically controlled temperature of 25 ± 0.5 °C. The values of the elongation growth and the pH of the incubation medium were sampled every 3 min using a multifunctional computer meter (CX-771; Elmetron, Zabrze, Poland).

### 4.3. Hydrogen Peroxide Detection

The hydrogen peroxide (H_2_O_2_) level in all of the treatments was determined according to Velikova et al. [74] and Junglee et al. [75], with minor modifications. Briefly, 0.5 g coleoptile segment samples were homogenised in 1.5 mL of 0.1% (*w*/*v*) trichloroacetic acid (TCA) and centrifuged at 10,000 rpm at 4 °C for 6 min. Subsequently, 0.5 mL of the supernatant was added to a solution containing 0.5 mL of a 0.1 M K-phosphate buffer and 1 mL of 1 M KI. After 1 h of incubation in the dark, the absorbance of the supernatant was measured at 350 nm. The content of H_2_O_2_ was calculated from a standard calibration curve that was prepared in 0.1% TCA at different H_2_O_2_ concentrations and is expressed as μmol/g fresh weight (FW). 

### 4.4. Redox Activity

To determine the redox activity, the coleoptile segments were prepared in the same manner as for the growth experiments, then preincubated for 1 h in distilled water and transferred to the solution of 1 mM Tris-HCl (pH 6.0) with 0.5 mM CaCl_2_, 50 mM KCl, 1 mM HCF III (ferricyanide), IAA and naphthoquinones at the appropriate concentration. The samples were shaken at 100 rpm and the level of HCF III reduction was measured every 30 min for the next 2 h. The redox activity, which was measured as the decay of the HCF III absorption at 420 nm and expressed in nmol of the reduced hexacyanoferrate III that was calculated per g of FW was previously described by Federico and Giartosio [76]. To avoid the influence of the presence of naphthoquinones in the incubation medium on the absorption of the light at 420 nm, we modified the values of the HCF III reduction by the same amount as the values that were recorded with no HCF III in the incubation medium. The results are the means of three independent experiments.

### 4.5. Lipid Peroxidation

Lipid peroxidation was determined by estimating the MDA content, which was determined as described by Hodges et al. [77], with minor modifications. The 0.5 g samples of coleoptile segments were immediately placed in liquid nitrogen and homogenised in 12.5 mL 80% ethanol. A 1 mL aliquot of a diluted sample was added to a test tube with 1 mL of either (1) a −TBA solution comprised of 20% (*w*/*v*) trichloroacetic acid and 0.01% butylated hydroxytoluene or (2) a +TBA solution containing the above plus 0.65% TBA. Afterwards, the samples were mixed vigorously, heated at 95 °C in a boiling water bath for 20 min, before being cooled and centrifuged at 10,000 rpm at 4 °C for 10 min. The absorbance values were read at 440, 532 and 600 nm. The MDA equivalents were calculated in the following manner:

[(Abs_532+TBA_)  −  (Abs_600+TBA_)  −  (Abs_532−TBA_  −  Abs_600−TBA_)] = A (1)

[(Abs_440+TBA_  −  Abs_600+TBA_) 0.0571] = B (2)

MDA equivalents (nmol/mL) = (A  −  B/157000) × 10^6^ (3)

### 4.6. Viability

The nonviable cells were visualised using the procedure described by Hunter et al. [78], with minor modifications. The samples of 1 cm coleoptiles after incubation for 10 h with the appropriate naphthoquinones under dim green light were transferred to a solution containing 0.1 mg/mL (0.15 mM) propidium iodide for 5 min at 25 °C. Afterwards, the samples were rinsed thoroughly in distilled water in order to remove any excess dye and placed on glass slides. For the visualisation, the samples were imaged using a Olympus IX81 Inverted Compound Microscope equipped with a FluoView FV1000 confocal system (Olympus, Waltham, United State). The viability was estimated as the percentage of living cells.

### 4.7. Statistical Analysis

The data were analyzed using Statistica software for Windows (STATISTICA data analysis software system, version 12.0 http://www.statsoft.com, USA) at a significance level of 0.05. A one-way ANOVA (Analysis of Variance) was used to examine the effect of the tested treatment on the endogenous or auxin-induced growth of maize coleoptile cells. Afterwards, the post hoc least significant difference (LSD) test was used (*p*  <  0.05) to note the dissimilarity between the tested treatments inside the adequate group (endogenous or auxin-induced growth). The Student’s *t*-test was performed to evaluate the significance of the differences between the effects of naphthazarin combined with lawsone or 1,4_naphthoquinone on the endogenous and IAA-induced growth.

## Supplementary Materials

Supplementary materials can be found at https://www.mdpi.com/1422-0067/20/7/1788/s1.

## Abbreviations

DHNQ	5,8-dihydroxy-1,4-naphthoquinone
NQ-2-OH	2-hydroxy-1,4-naphthoquinone
NQ	1,4-naphthoquinone
IAA	Indole acetic acid
