# Supplementary material for: Effects of Naphthazarin (DHNQ) Combined with Lawsone (NQ-2-OH) or 1,4-Naphthoquinone (NQ) on the Auxin-Induced Growth of Zea mays L. Coleoptile Segments
Source: Int J Mol Sci. 2019 Apr 11;20(7):1788. doi: 10.3390/ijms20071788 (PMC6479706; doi:10.3390/ijms20071788)
Supplement: Supplementary file 1 [file ijms-20-01788-s001.pdf]

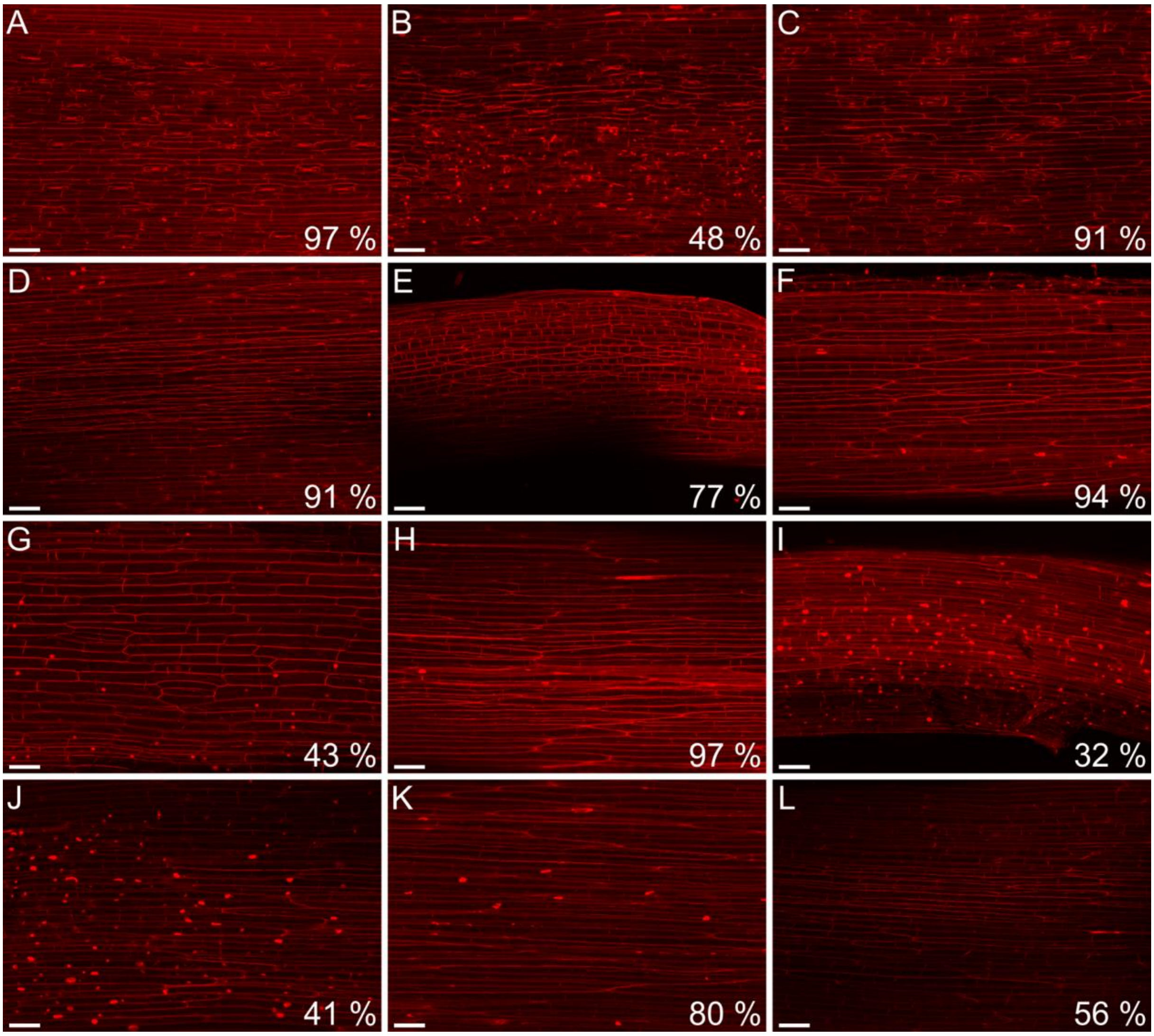

Supplementary Materials 1. The effect of DHNQ, NQ-2-OH and NQ on the membrane integrity and cell viability in the maize coleoptile segments. (A) 1 nM DHNQ, (B) 1 nM NQ-2-OH; (C) 1 nM NQ; (D) 10 nM DHNQ; (E) 10 nM NQ-2-OH; (F) 10 nM NQ; (G) 1 nM DHNQ + IAA; (H) 1 nM NQ-2-OH + IAA; (I) 1 nM NQ + IAA; (J) 10 nM DHNQ + IAA; (K) 10 nM NQ-2-OH + IAA; (L) 10 nM NQ + IAA. Cell viability is presented as the percentage of living cells.
